# Endoparasitic Insights of Free-Living Fin (*Balaenoptera physalus*), Humpback (*Megaptera novaeangliae*) and North Atlantic Right Whales (*Eubalaena glacialis*) from Eastern Canadian Waters

**DOI:** 10.1007/s11686-020-00298-9

**Published:** 2020-10-31

**Authors:** S. Kleinertz, L. M. R. Silva, S. Köpper, C. Hermosilla, C. Ramp

**Affiliations:** 1 Aquaculture and Sea-Ranching, Faculty of Agricultural and Environmental Sciences, University of Rostock, Justus-von-Liebig-Weg 2, 18059 Rostock, Germany; 2 Faculty of Fisheries and Marine Sciences, IPB University (Bogor Agricultural University), Jl. Agatis Kampus IPB Dramaga, Bogor, Indonesia; 3 Institute of Parasitology, Justus Liebig University Giessen, 35392, Schubert Str. 81, Giessen, Germany; 4 Mingan Island Cetacean Study, St. Lambert, QC J4P 1T3 Canada; 5 Sea Mammal Research Unit, Scottish Oceans Institute, University of St. Andrews, St. Andrews, KY16 8LB Fife UK; 6 University of Bremen, Bibliothekstr. 1, 28359 Bremen, Germany

**Keywords:** Zoonoses, Endangered mammal species, Conservation

## Abstract

**Purpose:**

To date, little is still known on parasite infections affecting free-living large whale populations worldwide. Data presented should be considered as a baseline study for future monitoring surveys on endoparasites affecting whales, thereby enhancing investigations on impacts of zoonotic parasitoses not only on vulnerable or endangered baleen whale population health but also on public health.

**Methods:**

The presented study is a first report on gastrointestinal parasites infecting different free-living baleen whales inhabiting East Canadian waters using non-invasive methods. Individual faecal samples from fin (*n* = 3; *Balaenoptera physalus*), humpback (*n* = 4; *Megaptera novaeangliae*) and North Atlantic right whales (*n* = 1; *Eubalaena glacialis*) were collected without animal disturbance, within their natural habitats on an ecological expedition during annual surveys in summer 2017. Faecal samples were assessed by standardized diagnostic methods, such as sodium acetate acetic formalin (SAF) technique, carbol fuchsin-stained faecal smears, *Giardia*/*Cryptosporidium* coproantigen ELISAs and were applied for further identification.

**Results:**

Parasitological infections included three different potentially zoonotic parasite species, one protozoa (*Entamoeba* spp*.*) and two metazoans (Diphyllobothriidae gen. sp., Ascaridida indet.). No positive *Giardia*/*Cryptosporidium* coproantigen ELISA could be found in the studied whales.

**Conclusion:**

This study adds to the current knowledge of intestinal and zoonotic parasite infections of vulnerable to partly endangered free-ranging baleen whales. Only few or no parasitological studies exist for these whale species, usually dealing with only one dead specimen. We call for more research in this field especially for the importance of conservation of free-living marine mammals using non-invasive methods.

## Introduction

Whale species, especially large whales are extraordinarily difficult to sample in the open ocean since they spend most of their time submerged, coming to the surface only for brief instants [1, 2] or migrating over enormous distances [3, 4]. According to Hermosilla *et al.* [1] large whales are of special public interest and have been subjected to a variety of conservation measures, which could be better monitored and managed if physiological and pathophysiological data, such as parasite infections, could already be gathered from wild and free-ranging animals, instead of carcasses, within future studies [5]. Moreover, there are still no facilities for detailed examinations to accommodate whale species larger than ~ 8 m of length [e.g., larger than killer whales (*Orcinus orca*)] in captivity which seriously hampers application of most classical parasitological diagnostic methods [2, 5]. Consequently, the present knowledge of diseases of large whales is still very scarce when compared to terrestrial vertebrate taxa [6].

Listed as endangered is the North Atlantic right whale (*Eubalaena glacialis*) under the Endangered Species Act (ESA) in 2005 [7] (also see IUCN Red List). Commercial whaling has severely depleted these whale species populations during past decades. More recently, direct and indirect anthropogenic impacts, namely in the form of vessel collisions and entanglement in fishing gear, have accounted for a lack of recovery [7]. With roughly 400 individuals still alive, the western North Atlantic population of right whales is one of the most critically endangered and vulnerable of any whale population in the world [8–10]. Within two centuries, this population could face extinction [11]. Therefore, Doucette *et al*. [12] as well as Fisheries and Oceans Canada [13] mentioned reports by the International Whaling Commission [14] and an expert panel [15], which strongly recommend, amongst others, to investigate the role of reproductive failure and declining health in impeding recovery of *E. glacialis*. Fin (*Balaenptera physalus*) and humpback whales (*Megaptera novaeanglia*) are listed differently from “least concern” to “vulnerable”, respectively [16, 17].

Parasitic diseases are increasingly recognized for their profound influences on individual, population, and even ecosystem health [1, 6]. In fact, little is known about endo- and ectoparasites of any wild cetacean population [1, 6, 18–20]. In recent years, ´Emerging Infectious Diseases´ (EIDs) [21] have been reported in several cetacean species and populations worldwide provoking large-scale die-offs, affecting reproduction, causing disfiguring skin diseases and, in some cases, zoonoses [22].

Within this study, large baleen whale species including fin whales, humpback whales and North Atlantic right whales were examined for their gastrointestinal parasites using non-invasive methods as reported elsewhere [1, 2, 5, 6, 19].

The present study aimed to identify gastrointestinal parasites of vulnerable or even endangered free-ranging marine mammal species like fin and North Atlantic right whales as well as to support the population status of least concerned humpback whales within their natural habitats by analyzing faecal samples, using non-invasive methods first introduced by Kleinertz *et al*. [20], in the North Atlantic Ocean to shed light on the health status of these protected and partly endangered large marine animals.

## Materials and Methods

The Mingan Island Cetacean Study (MICS)  examines baleen whales in the Gulf of St. Lawrence since 1979, the main research is the Jacque Cartier Passage (49° 54 41 N–64° 32 01 W). The Jacques Cartier Passage is located between the Quebec North Shore and Anticosti Island, the research area extends a bit further to the east and west, it is approximately between 63.0 and 66.0° West and and between 49.6 and 50.3° North. For this study, MICS collected faeces samples of fin, humpback and North Atlantic right whales *(Balaenoptera physalus, Megaptera novaeangliae, Eubalaena glacilis*), in July and August of 2017. All research was conducted under the approval of the Animal Welfare and Ethics Committee (AWEC) of the School of Biology, University of St. Andrews, UK.

**Fig. 1:**
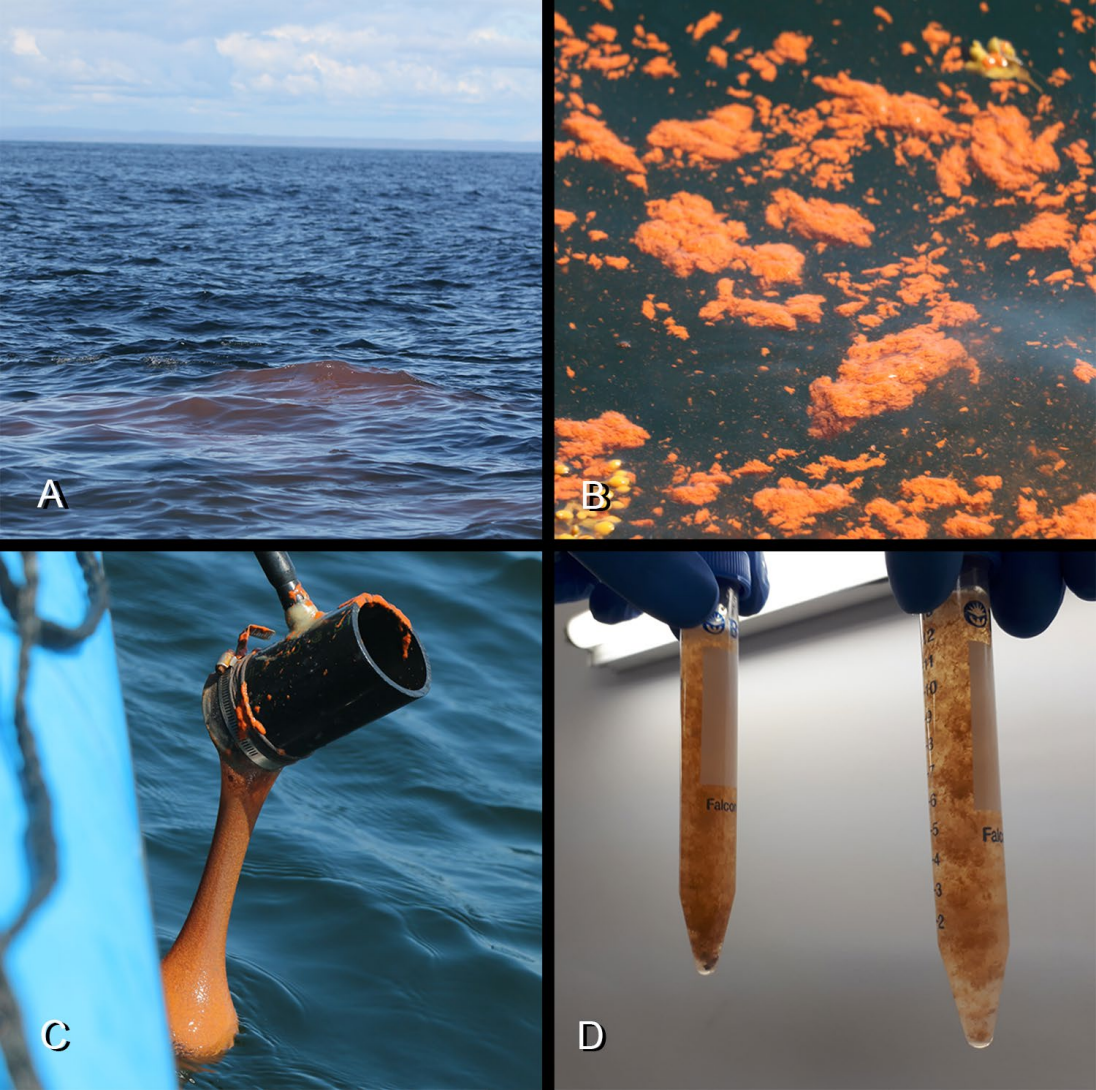
Faecal sample collection: **a**, **b**: Fresh whale faeces on the ocean surface, **c**: Collection of faecal sample with a nylon device by boat, **d**: Preparation of faecal samples for analysis with ethanol

Faecal samples were collected opportunistically. Inflatable boats approached individuals or a group of whales for photo identification. This procedure enabled the detection of faecal material floating on the surface when an animal defecated during its surfacing time (Fig. 1a, b). A self-built device consisting of an extendable handle bar and a piece of PVC pipe was used to collect the samples (Fig. 1c). At one end of the pipe, a piece of nylon stocking was attached with a hose clamp. This faeces collection device was used to amass as much material as possible. The device was lifted out of the ocean to lose most of the excess water (Fig. 1c). The filled nylon stocking was then placed in two zip lock bags to prevent leakage and stored on ice packs in a cooler. Back in the lab, the material from the nylon stocking was removed directly using a spatula. 25 ml of the faecal sample was transferred into two 15 ml tubes, which were topped up with 70% and 90% ethanol, respectively (Fig. 1d).

Coproscopical analyses were performed at Institute of Parasitology, Justus Liebig University Giessen, Germany, using the standard sodium acetate acetic acid formalin (SAF) technique with ethyl acetate [23, 24]. The SAF technique was used for the detection of parasite eggs, cysts, sporocysts and oocysts within faecal material. Furthermore, coproantigen ELISAs (ProSpecT^®^, Oxoid) were performed for the detection of *Cryptosporidium* spp. and *Giardia* spp. antigens in faecal samples. The parasitological identification of eggs was based on morphological characteristics referring to other reports and original parasite descriptions [25, 26]. Parasitological calculations (prevalence in %) were made according to Bush *et al*. [27].

## Results

Four out of eight examined whales proved to be parasitized (50%). Parasitological analyses of faecal samples of free-ranging baleen whale species revealed three different parasites: one (neozoan) protozoan taxa (*Entamoeba* spp.) and two metazoan parasite taxa (Cestoda: *Diphyllibothrium* cf. *balaenopterae*, Nematoda: Ascaridida indet.). [*Entamoeba* spp. (50% prevalence), *Diphyllobothrium* spp. (25% prevalence) and Ascaridida indet. (25% prevalence)].

Neither trematode nor acantocephalan eggs were detected by microscopical examination of the SAF concentrated faecal samples. Furthermore, all samples proved negative for *Cryptosporidium* spp. and *Giardia* spp. performing coproantigen-ELISAs.

## Discussion

During the past years, investigations on intestinal parasites of cetacean species usually rely on accidental strandings of single or small numbers of animals, on animals in captivity living or on dead specimens obtained from marine zoos [28]. By obtaining faecal samples directly from wild and free-ranging large whale species, these novel surveys bring the potential to unveal unique insights into the actual gastrointestinal parasite fauna of live, wild large whales ranging free within their natural environments [5, 20].

Due to the difficulties to obtain further appropriate samples, within the current study relatively low amount of faecal samples is considered. Nevertheless, all isolated parasite taxa within this study have zoonotic potential, even though presenting low prevalences. All detected parasite taxa have been reported already for fin whales [1, 2], but not for the other species studied.

*Entamoeba* spp. infections in whale species have been only reported by Heckmann *et al*. [29] and Raga *et al*. [30] for dead bowhead whales, and by Hermosilla *et al*. [2] for free-ranging blue, fin and sei whales from the Azores, with relatively high prevalences (64.7%). According to Hermosilla *et al*. [2], besides several non/low pathogenic species, such as *Entamoeba coli*, *E. hartmanni, E. suis, E. polecki*, and *Iodamoeba bütschlii* [31], there are species with high pathogenic and/or zoonotic relevance like *E. histolytica*.

In this study, we detected Diphyllobothriidae gen. sp. eggs (most likely *Diphyllobotrium* cf. *balaeonopterae*) in humpback whale faecal samples (50% prevalence in humphback whale samples). *Diphyllobotrium* spp. infections in cetaceans are usually harmless [32, 33], but in cases of high parasitic burdens, weakening and even death of parasitized hosts may be observed [20].

Ascaridida indet. eggs were detected in all four humpback whale faecal samples (overall prevalence of 50%), but a morphological characterization to species level was not possible, since the eggs typify members of different genera, such as *Anisakis*, *Pseudoterranova* or *Contracaecum.* These genera have been reported frequently in marine mammals, which act as definite hosts to these species [34–36]. The ascarid nematode eggs most likely represent the genus *Anisakis,* given that the other genera mentioned rarely parasitize cetaceans [26] nematodes of the genera *Anisakis* are known to infect cetaceans as definite hosts [37].

In conclusion, this study adds to the current knowledge of intestinal parasite infections of vulnerable to partly endangered free-ranging baleen whales. Only few or no parasitological studies exist for these whale species, usually dealing with only one specimen, often from more or less degenerated carcasses [28, 38]. We call for more research in this field especially for the importance of conservation of free-living marine mammals, like whales in marine ecosystems under threat and for monitoring reasons of marine mammal health, encouraging long-term, worldwide and joint interdisciplinary sample efforts.

## Data Availability

Not applicable.
